# Supplementary figures and images for: Unpredictability of the “when” influences prediction error processing of the “what” and “where”
Source: PLoS One. 2022 Feb 3;17(2):e0263373. doi: 10.1371/journal.pone.0263373 (PMC8812910; doi:10.1371/journal.pone.0263373)

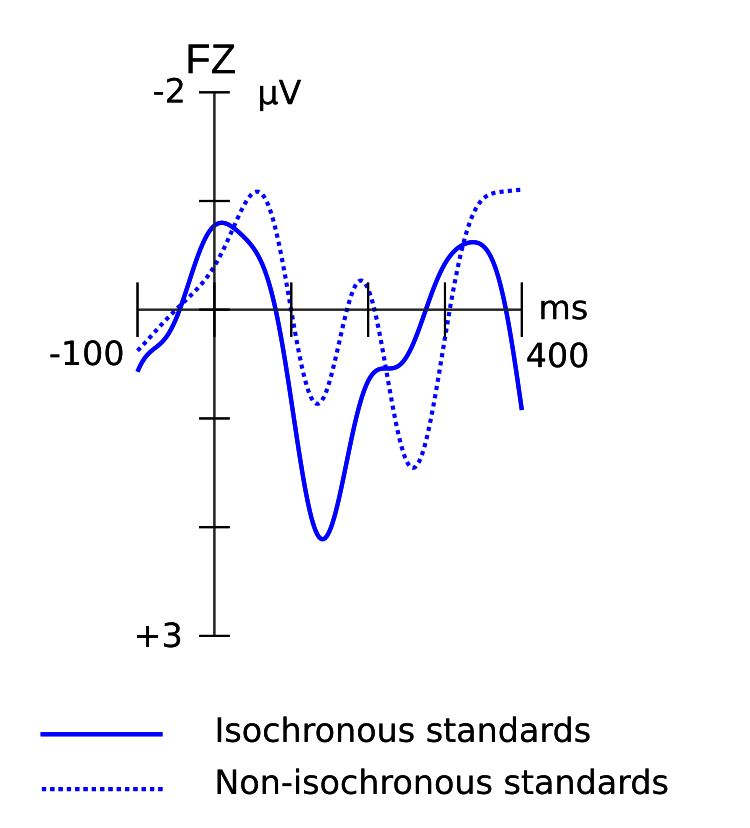

Supplement: S1 Fig — Mean ERP waves for standards (high-probability) endings under non isochronous and non-isochronous stimulation as captured at electrode Fz. (TIFF) [file pone.0263373.s001.tiff]
