# Supplementary material for: Unpredictability of the “when” influences prediction error processing of the “what” and “where”
Source: PLoS One. 2022 Feb 3;17(2):e0263373. doi: 10.1371/journal.pone.0263373 (PMC8812910; doi:10.1371/journal.pone.0263373)
Supplement: Attachment — Submitted filename: Response to Reviewers.pdf [file pone.0263373.s003.pdf]

Response to the editor's and reviewers' comments

Journal Requirements:

Comment 1. The response to the data sharing requirement is incomplete. There are ways to make measured data unidentifiable: please consult your ethical committee. The analysis scripts and code could also be shared.

Response:

We agree that in our submitted version we were not able to satisfy your request to make the data publicly available. In the new version of our manuscript we provide a link to our data which we made them unidentifiable.

Response to Reviewer comments:

Comment 1: The authors addressed all my comments. I have only few minor suggestions for tables and figures.

Response: *We are very grateful to the useful suggestions made by the reviewer. In our revised paper we have made all the necessary changes to address all the reviewer's remarks regarding the tables, the figures and the text.*
Specifically, in the tables:
- we have replaced references to the "previous study" with ("Tsogli et al, 2019").
- we have added the SOA range, the 'real' interstimulus interval. Additionally on the Table legend we have added definitions for the SOA and interstimulus interval to avoid confusion:

> *Interstimulus interval denotes the silent interval between the offset of one tone and the onset of the next one. SOAs denote the interval between the onset of one tone and the onset of the next one*

- on Table 4, we have replaced "current study" with "Tsogli et al. 2019" (under box: Isochronous stimulation).

In the figures:
- we have corrected the last example triplet of the train from CDE to CDF.
- we have changed the legend title of Figure 2 so that it is clear to the reader that the depicted results pertain to two different experiments/studies. The new legend title follows:

> *ERPs of statistical and physical deviants as recorded in the current study with non-isochronous sound presentation, and in the previous study [25] with isochronous sound presentation.*

- we have modified the legend of Figure 2 to denote that the shaded area on the ERPs represent SEM. The new legend follows:

> *The shaded area on the ERPs represents the SEM.*

In the text:
- We have modified line 342 of the manuscript where we mistakenly wrote "EPRs" instead of "ERPs".
